# Meningococcal Vaccines: Challenges and Prospects

**DOI:** 10.3390/vaccines8040738

**Published:** 2020-12-04

**Authors:** Priyal Bagwe, Lotika Bajaj, Rikhav Gala, Susu M. Zughaier, Mohammad N. Uddin, Martin J. D’Souza

**Affiliations:** 1 Center for Drug Delivery Research, Vaccine Nanotechnology Laboratory, College of Pharmacy, Mercer University, Atlanta, GA 30341, USA; Priyal.Bagwe@live.mercer.edu (P.B.); Lotika.Bajaj@live.mercer.edu (L.B.); Uddin_mn@mercer.edu (M.N.U.); 2 Fraunhofer USA Center for Molecular Biotechnology, Newark, DE 19711, USA; rikhav.praful.gala@gmail.com; 3 College of Medicine, QU Health, Qatar University, Doha P.O. Box 2713, Qatar; szughaier@qu.edu.qa

*Neisseria meningitidis* is a gram-negative bacterium that causes a severe acute infection, called the meningococcal disease. Meningococcus continues to impact public health and is the root cause of meningitis, sepsis, and a focal disease (e.g., pneumonia). According to the World Health Organization (WHO), the meningococcal disease is associated with a high fatality rate of up to 50%, if untreated [1]. The field of meningococcal disease is continuously growing with exciting developments in vaccine generation; including polysaccharide, conjugate, glycoconjugate, and protein-based vaccines. This issue features recent research discoveries that may help develop vaccine studies for meningococcal disease in the future.

The virulent capsular polysaccharide of meningococcus forms the basis of preventive vaccines. Out of the 12 identified disease-causing serogroups (based on composition of capsular polysaccharides) of *N. meningitidis*, 6 serogroups: A (sub-Saharan Africa), B (Europe and North America), C (Europe and North America), W (Africa and South America), X (Africa) and Y (Europe and North America) cause the disease [2]. Out of these 6 serogroups, 5 serogroups: A, C, W and Y (polysaccharides and glycoconjugate) and B (protein-based) have effective vaccines [3]. Currently, there are no vaccines for serogroup X.

Polysaccharide, as vaccine antigens are thymus independent thereby generate short-lived T-cell-independent immune response, with an age-dependent response. These polysaccharide vaccines have poor immunogenicity in children younger than 2 years of age, with little boost in antibody titers following booster doses and are generally used during epidemics [1]. The antibody responses in polysaccharide quadrivalent vaccines are serogroup specific and independent (no cross protection). Mencevax^®^ (GlaxoSmithKline Biologicals, Rixensart, Belgium), is commercially available in Europe while Menoumne^®^ (Sanofi Pasteur Inc., Swiftwater, PA, USA), was available in the United States until 2017 (discontinued) [2]. Conjugate vaccines elicit immunologic memory responses [1]. Two single component conjugate vaccines, Menactra^®^—MenACWY (Sanofi Pasteur Inc., Swiftwater, PA, USA) and Menveo^®^ - MenACWY-CRM (GSK Vaccines, Srl, SI., Italy), and one multi-component vaccine in combination with *Hemophilus influenza b* (Hib), MenHibrix^®^—Hib-MenCY-TT (GlaxoSmithKline Biologicals, Rixensart, Belgium), are also licensed in the United States. Routine vaccinations (once every 5 years) are recommended for conjugate vaccines specially for children who have been immunized and remain at risk.

Protein-based vaccines against serogroup B meningococcal disease, Bexsero^®^ (GSK Vaccines, Srl, SI., Italy)and Trumenba^®^ (Wyeth Pharmaceuticals Inc., Philadelphia, PA, USA), are available in the United States. The four-component vaccine Bexsero^®^, contains NHBA (a recombinant *Neisseria* heparin binding antigen), NadA (recombinant *Neisseria* adhesin A), fHbp (a recombinant complement factor H binding protein) and Porin A [4]. Trumenba^®^, a two-component vaccine, contains two lipidated antigenic variants of fHbp [5]. Both Bexsero^®^ and Trumenba^®^ have demonstrated ≥ 4-fold immune response against three and four different strains of *N. meningitidis* respectively. The outer membrane vesicle (OMV)-based serogroup B vaccine in combination with polysaccharide from serogroup C, VA-MENGOC-BC^®^ (Finlay Institute, Havana, Cuba) has significantly reduced the burden of disease in Cuba [1]. The conventional production methods for meningitidis glycoconjugate vaccines involve attaching the oligosaccharide component to the carrier proteins. They have several disadvantages such as exposure to the disease-causing bacteria and improper carbohydrate-protein conjugations.

The current research is focused on chemical and chemoenzymatic synthesis of the polysaccharides to better control the quality of carbohydrate being produced. These polysaccharides are then conjugated to protein carriers forming glycoconjugate vaccines. The oligosaccharides of different chain lengths, serogroup W, serogroup A, and serogroup C are few published studies involving chemical synthesis [6]. The chemoenzymatically synthesized, genetically modified form of serogroup C and serogroup X oligosaccharides are produced and conjugated with carrier proteins [6]. The immunogenicity of novel carrier proteins from different types of bacteria against *N. meningitidis* is being investigated. Lipopolysaccharides (LPS) and OMVs are potential targets for a broad vaccine candidate. Genetic modification, deletion of certain genes from LPS might result in production of OMV with reduced toxicity. Different proteins like the porins- Por A & Por B, the adhesin & adenosine triphosphate (ATP)-binding cassette transporters have been explored as novel targets for an effective protective vaccine [7,8]. Additionally, nanoparticulate delivery of vaccine antigen via encapsulation of protein antigen, or fusion of the antigen with heterodimers of VipA-VipB proteins, offers a new area of research [6,9,10]. In recent pre-clinical reports, novel vaccine delivery systems such as oral dissolving films, osmotic releasing buccal tablets, and microneedle based transdermal systems are being evaluated for improving the patient compliance of immunizations [9,11].

Future work in vaccine development seeks on discovering novel targets such as proteins, LPS and OMVs. One critical component of homogenous vaccines are carrier proteins. The potential of novel carrier proteins to produce safe and effective vaccine candidates can be investigated. Use of site-specific conjugation methods for known carrier proteins will help in developing homogenous vaccines. Another challenge in laboratory synthesis, is the O-acetylation of polysaccharide groups which causes increased virulence and decreased antigen presentation in serogroups A, B, C, W and Y. Similarly, chemoenzymatic synthesis would require developing O-acetyltransferases and enzymes producing capsules [6]. These processes are quite time-consuming as there is a need to develop the exact epitope that would bind specifically to the serogroup and produce bactericidal antibodies. Optimizing the scale-up of these laboratory synthesis methods of the polysaccharide antigens is another hurdle in developing effective vaccines.

Advancements in carbohydrate microarray technology could be applied for the development of meningococcal vaccine. Further studies built on the foundations of solid phase chemical synthesis, enzymatic and chemoenzymatic synthesis are required for vaccine development.

Vaccine efficacy is a measure of reduction in infection incidences among vaccinated individuals and marked reduction in the burden of disease at the community level. In order to reliably predict the vaccine efficacy clinically there is a need for surrogate markers of protection. One of the critical mechanisms of immunity against meningococci is activation of the complement cascade system. It has been demonstrated that serum bactericidal antibodies i.e., complement-mediated killing, triggered by meningococcal vaccines offer protection. However, the performance of serum bactericidal activity (SBA) assays are dependent on several factors like source of complement, duration of immune protection (immediate, long-term; SBA titer), and asymptomatic colonization. Other potential surrogates of protection would be to measure opsonophagocytic activity, whole-blood cell activity, immunoassays, vaccine response in complement-deficient individuals. These assays will help develop an ideal vaccine which would provide long-lasting protection against wide-range of serogroups in complement-deficient individuals and help improve the sensitivity of vaccine efficacy testing [12].

Homogenous vaccine strategies with well-defined antigen immunogenicity will likely follow the novel discoveries (chemical and chemoenzymatic synthesis routes). With a rise in the prevalence of meningococcal disease serogroup X, there is a need for an effective vaccine against serogroup X. Further research for developing affordable vaccines to be used in developing countries must be pursued.
